# Optogenetic Activation of Arcuate Kisspeptin Neurons Generates a Luteinizing Hormone Surge-Like Secretion in an Estradiol-Dependent Manner

**DOI:** 10.3389/fendo.2021.775233

**Published:** 2021-11-02

**Authors:** Xian-Hua Lin, Geffen Lass, Ling-Si Kong, Hui Wang, Xiao-Feng Li, He-Feng Huang, Kevin T. O’Byrne

**Affiliations:** ^1^ Obstetrics and Gynecology Hospital, Institute of Reproduction and Development, Fudan University, Shanghai, China; ^2^ Department of Women and Children’s Health, Faculty of Life Sciences and Medicine, King’s College London, London, United Kingdom; ^3^ Department of Obstetrics and Gynecology, Maternal and Child Health Hospital of Songjiang District, Shanghai, China

**Keywords:** LH surge, gonadal steroids, arcuate kisspeptin, AVPV kisspeptin, optogenetics

## Abstract

Traditionally, the anteroventral periventricular (AVPV) nucleus has been the brain area associated with luteinizing hormone (LH) surge secretion in rodents. However, the role of the other population of hypothalamic kisspeptin neurons, in the arcuate nucleus (ARC), has been less well characterized with respect to surge generation. Previous experiments have demonstrated ARC kisspeptin knockdown reduced the amplitude of LH surges, indicating that they have a role in surge amplification. The present study used an optogenetic approach to selectively stimulate ARC kisspeptin neurons and examine the effect on LH surges in mice with different hormonal administrations. LH level was monitored from 13:00 to 21:00 h, at 30-minute intervals. Intact Kiss-Cre female mice showed increased LH secretion during the stimulation period in addition to displaying a spontaneous LH surge around the time of lights off. In ovariectomized Kiss-Cre mice, optogenetic stimulation was followed by a surge-like secretion of LH immediately after the stimulation period. Ovariectomized Kiss-Cre mice with a low dose of 17β-estradiol (OVX+E) replacement displayed a surge-like increase in LH release during period of optic stimulation. No LH response to the optic stimulation was observed in OVX+E mice on the day of estradiol benzoate (EB) treatment (day 1). However, after administration of progesterone (day 2), all OVX+E+EB+P mice exhibited an LH surge during optic stimulation. A spontaneous LH surge also occurred in these mice at the expected time. Taken together, these results help to affirm the fact that ARC kisspeptin may have a novel amplificatory role in LH surge production, which is dependent on the gonadal steroid milieu.

## Introduction

Normal reproductive function in mammals is controlled by the hypothalamic-pituitary-gonadal axis. The final common pathway for central neural regulation of reproduction consists of gonadotropin releasing hormone (GnRH) neurons, driving pulse and surge release of luteinizing hormone (LH) from the pituitary gland. In all mammalian species, the pre-ovulatory LH surge is induced by estrogen although the underlying mechanisms remain to be fully elucidated (1, 2).

A crucial activator of the GnRH network is Kisspeptin-encoded by the KISS1 gene (3). Inactivating mutations of KISS1 or its receptor lead to hypogonadotropic hypogonadism with a consequent failure to progress through puberty in humans and rodent models (4–6). Key populations of Kiss1 neurons in rodents are located in the anteroventral periventricular (AVPV) and arcuate nucleus (ARC) of the hypothalamus (7–9). Unlike GnRH neurons, both ARC and AVPV kisspeptin neurons express estrogen receptor (ER)-α, therefore acting as relay afferents for feedback. Of these populations, AVPV Kiss1 neurons have been noted to play a significant role in mediating positive estradiol feedback, leading to induction of the pre-ovulatory LH surge (10), while neurons in the ARC are a major component of GnRH pulse generation. These ARC neurons also co-express neurokinin B (NKB) and dynorphin A (Dyn) so are therefore referred to as KNDy neurons (11–13). These neurons innervate GnRH neurons in the preoptic area (POA) and median eminence to modulate GnRH release directly (14–16). However, the role of ARC Kiss1 in LH surge generation has been less well characterized. It has been demonstrated that selective ablation of KNDy neurons increased the magnitude of LH surges in steroid primed ovariectomized (OVX) rats; this was attributed to lack of inhibitory Dyn from KDNy neurons to those in the AVPV (17). Additionally, knockdown of kisspeptin in the ARC has been shown to decrease the amplitude of the LH surge (18). It has also been found that optogenetic activation of Kiss1 neurons of the rostral periventricular area of the third ventricle neurons *in vivo* at 10 Hz generated substantial increments in LH secretion of similar amplitude to the endogenous LH surge (19). With these results in mind, it is reasonable to propose ARC Kiss1 neurons may be involved in generating the pre-ovulatory GnRH/LH surge under optimal hormonal conditions.

In this study, we have used selective optogenetic activation of ARC Kiss1 neurons to examine their LH surge generating abilities and whether these responses are altered depending on sex steroid milieu.

## Materials and Methods

### Animals

Breeding pairs of Kiss-Cre heterozygous transgenic mice (20) were obtained from the Department of Physiology, Developmental and Neuroscience, University of Cambridge, UK. Litters from the breeding pairs were genotyped by polymerase chain reaction (PCR) analysis. Kiss-Cre (Cre+) and wild type (Cre-) female mice, experiencing normal pubertal development, confirmed by assessing normality of the estrous cycles and weighing between 25-30 g, were included in the study. Mice were kept singularly housed under controlled conditions (12 :12 h dark/light cycle, on at 07:00 h, 25°C) and provided with food and water *ad libitum*. All animal procedures were performed in accordance with the UK Home Office Regulations, approved by the Animal Welfare and Ethical Review Body Committee at King’s College London.

### Stereotaxic Injections of Adeno-Associated Virus

All surgical procedures were performed under aseptic conditions. General anesthesia was achieved using ketamine (Vetalar, 100 mg/kg, i.p.; Pfizer, Sandwich, UK) and xylazine (Rompun, 10 mg/kg, i.p.; Bayer, Leverkusen, Germany). Adult female mice were secured in a motorized Kopf stereotaxic frame and surgical procedures were performed using a robot stereotaxic system (Neurostar, Tubingen, Germany). A small hole was drilled in the skull at a location above the ARC. Using a 2-μL Hamilton micro-syringe (Esslab, Essex, UK) attached to the stereotaxic frame micro-manipulator, 0.4 μl of the ChR2 virus, AAV9-EF1a-double floxed-hChR2(H134R)-EYFP-WPRE-HGHpA (≥1×10^13^ vg/mL; Addgene, Massachusetts, USA) was injected unilaterally into the right ARC (−1.94 mm AP, 0.2 mm ML, 5.8 mm DV) over 10 min. The needle was left in position for a further 5 min and then removed slowly over 1 min. A fiber optic cannula (200 µm, 0.39NA, 1.25 mm ceramic ferrule; Thorlabs LTD, Ely, UK) was then inserted at the same co-ordinates as the injection site, but to a depth of 4.85 mm, so that the fiber optic cannula was situated immediately above the latter. Dental cement (Super-Bond Universal Kit, Prestige Dental, UK) was then used to fix the cannula in place, and the skin incision closed with suture. All mice, both Kiss-Cre (Cre+) (n = 30) and wild type (Cre-) (n = 5), received the AAV injection and implantation of a fiber optic cannula. Following a 1-week recovery period from surgery, the mice were handled daily to acclimatize them to the tail-tip blood sampling procedure.

### Gonadal Steroid Milieu

In addition to the gonadal intact (Cre+) (n = 10) and wild type (Cre-) (n = 5), a separate group of Cre+ mice were bilateral ovariectomy (OVX) at the same time as they received the AAV injection. Of the OVX animals, one group was implanted with a Silastic capsule (inner diameter: 1mm, outer diameter: 2.1 mm, Sanitech, Havant, UK) containing sesame oil as control (Sigma Chemicals Ltd., Poole, UK) (n = 6). The other group was implanted with a Silastic capsule containing 0.625 mg of estradiol (E) (Sigma) suspended in sesame oil (Sigma) at a concentration of 20 µg/ml (n = 14). These capsules produce circulating concentrations of E within the range observed during the diestrous phase of the estrous cycle (21). These animals were implanted with the E filled capsule (OVX+E) six days before optic stimulation to ensure stable E levels in the circulation. Seven to ten days later, the OVX+E mice received a s.c. injection of estradiol benzoate (EB) (1 µg / 20 g body weight in 0.05 ml sesame oil) at 09:00 h (OVX+E+EB), and 24 h later (the next day) a s.c. injection of progesterone (P: 500 µg in 0.05 ml of sesame oil) at 09:00 h (OVX+E+EB+P) to induce an LH surge.

### Optogenetic Activation *In Vivo*

Experiments were carried out at least 4 weeks following AAV injection, to ensure sufficient opsin expression as well as to allow for a sufficient habituation period. Prior to optogenetic stimulation, the very tip of the mouse’s tail was excised using a sterile scalpel for subsequent blood sample collection (21). The chronically-implanted fiber optic cannula was attached *via* a ceramic mating sleeve to a multimode fiber optic rotary joint patch cables (Thorlabs), allowing freedom of movement of the animal, for delivery of blue light (473 nm wavelength) using a Grass SD9B stimulator controlled DPSS laser (Laserglow Technologies, Toronto, Canada). Laser intensity at the tip of the fiber optic patch cable was 5 mW. After 1 h acclimatization, serial 4-μL tail blood samples were taken every 30 min from 13:00 - 21:00 h. Mice that received sustained optic stimulation (5-ms pulses) at frequencies of 5 Hz for 1.5 h did so between 14:30 - 16:30 h (early) and/or 18:00 - 19:30 h (late). The OVX mice received both the early and late stimulation protocols assigned in a random order. The OVX mice with steroid replacement (OVX+E, OVX+E+EB and OVX+E+EB+P sequentially) were randomly assigned to 2 separate groups (n = 7 per group) and exposed to different stimulation protocols or no stimulation. At least 3 days were allowed between experiments.

### LH Assays

A sensitive sandwich ELISA for the assessment of whole blood LH concentrations was used according to the protocol described by Steyn et al. (22). A 96 well ELISA plate (NUNC 96) was coated with coating antibody (1:1000, LHβ 518B7, University of California, Davis, California USA) and incubated overnight at 4°C. Mouse LH standard and antibody were purchased from Harbour-UCLA (California, USA). Secondary antibody (NA934) was from VWR International (Leicestershire, UK). The intra assay and inter-assay variations were 4.6% and 10.2%, respectively.

### Validation of Injection Site

After completion of experiments, mice were anaesthetized with a lethal dose of ketamine and transcardially perfused with heparinized saline for 5 min, followed by 10 min of ice-cold 4% paraformaldehyde (PFA) in phosphate buffer (pH 7.4) for 15 min using a pump (Minipuls, Gilson, Villiers Le Bel, France). Brains were rapidly collected and postfixed sequentially at 4°C in 15% sucrose in 4% PFA and in 30% sucrose in phosphate-buffered saline until they sank. Afterwards, brains were snap-frozen on dry ice and stored at -80°C until processing.

Brains were coronally sectioned (30-µm) using a cryostat (Bright Instrument Co., Luton, UK) and every third section was collected between -1.34 mm to -2.70 mm from the bregma. Sections were mounted on microscope slides, air-dried and cover slipped with ProLong Antifade mounting medium (Molecular Probes, Inc. OR, USA). The injection site was verified and evaluated and only animals expressing EYFP fluorescent protein in the ARC were included in the analysis by using Axioskop 2 Plus microscope equipped with Axiovision, version 4.7 (Zeiss).

### Statistical Analysis

The effect of optogenetic stimulation on circulating levels of LH was analyzed by comparing the mean levels of LH before (13:00 - 14:30 h), during (14:30 -16:30 or 18:00 - 19:30 h) and after (16:30 - 18:00 h or 19:30 - 21:00 h) stimulation. Analysis was also undertaken by calculating the area under the curve before and during LH surge-like release, or at different experimental periods, e.g., before (AUC_b_, 13:00 - 14:30 h), during (AUC_st_, 14:30 - 16:30 or 18:00 - 19:30 h) and after stimulation (AUC_a_, 16:30 - 18:00 h or 19:30 - 21:00 h). The time window for calculating the AUC for spontaneous LH surges was selected appropriately to reflect their actual time of day and duration. Comparisons between groups were made by subjecting data to one-way ANOVA followed by Dunnett’s test. SPSS (version 19.0 for windows) was used for the statistical analysis. Data are presented as the means ± SEM, and P < 0.05 was considered statistically significant.

## Results

### Anatomical Localization of Enhanced Green Fluorescent Protein After ARC Injection

**Figure 1:**
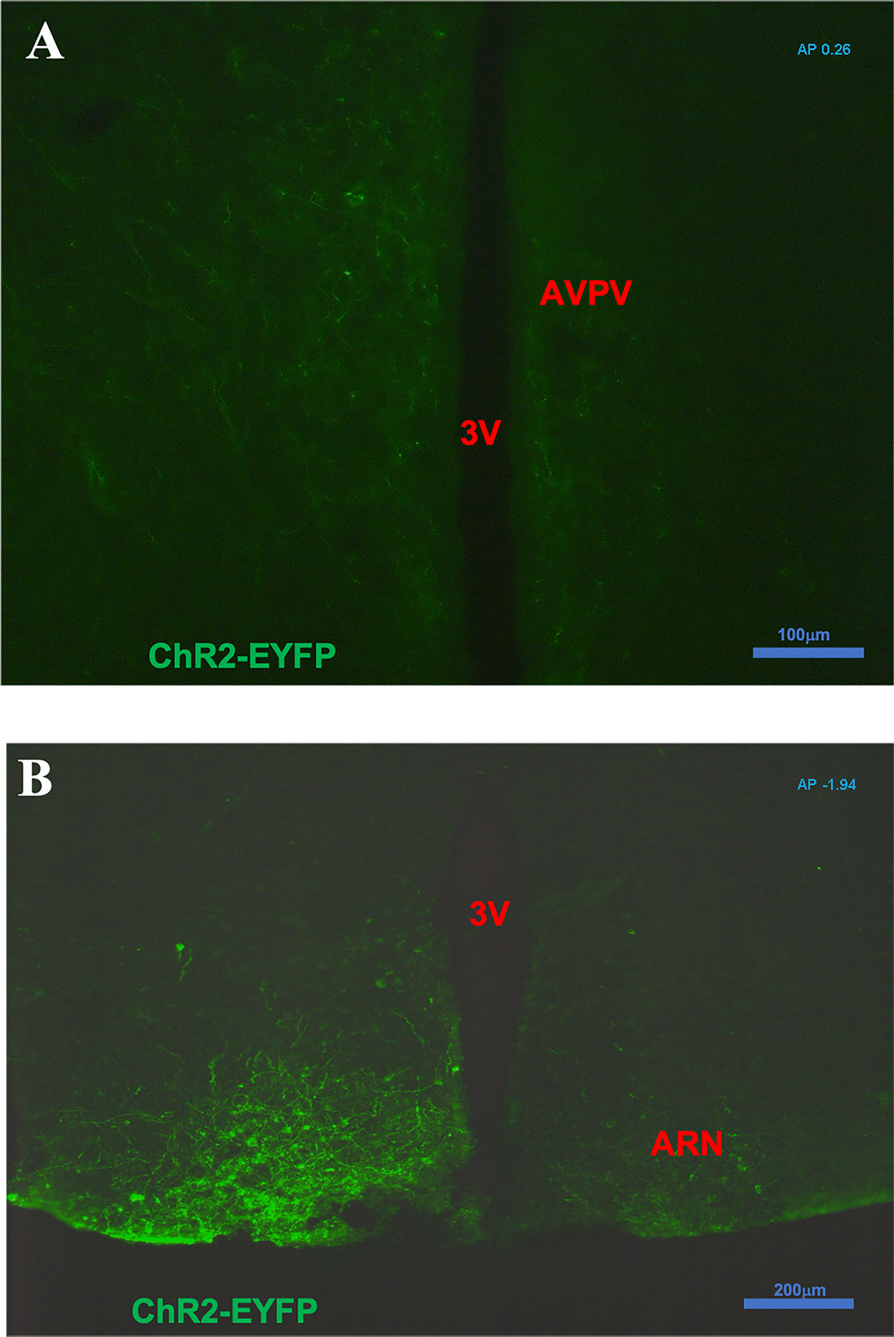
Expression of ChR2-EYFP in kisspeptin neurons in the arcuate nucleus (ARC) and fibers in the anteroventral periventricular nucleus (AVPV) in Kiss-cre mice. The coronal brain sections were obtained from Kiss-CRE mice to verify the expression of ChR2-EYFP in Kisspeptin neurons. **(A)** Note a number of ARC Kiss1 projection fibers showing in the AVPV (AP +0.26 from Bregma); especially obvious on the ipsilateral virus injection side. **(B)** Note kisspeptin neurons with ChR2-EYFP expression in the virus (pAAV-EF1a-double floxed-hChR2(H134R)-EYFP-WPRE-HGHpA) injection side of ARC area (AP -1.94 from Bregma).

After intra-ARC AAV9-EF1a-double floxed-hChR2(H134R)-EYFP-WPRE-HGHpA injection, EYFP-containing cell bodies and axons were observed at high density within the ARC, without spread to surrounding hypothalamic nuclei (**Figure 1**). A number of Kiss1 (ARC) projection fibers were seen in the AVPV, especially obvious on the ipsilateral virus injection side (**Figure 1A**). Analysis of images acquired from coronal sectioning of the mouse brains showed that 24 out of the 30 Cre+ animals had successful stereotaxic injection of AAV-ChR2 virus into the ARC (Gonadal intact: 8 out of 10; OVX: 5 out of 6; OVX with steroid replacement: 11 out of 14). Four out of 5 wild type (Cre-) had correct placement of the AAV-ChR2 virus.

### LH Surge Profiles and Effect of Optogenetic Stimulation of ARC Kiss1 Neurons on LH Secretion in Ovary Intact Mice on the Day of Proestrus

**Figure 2:**
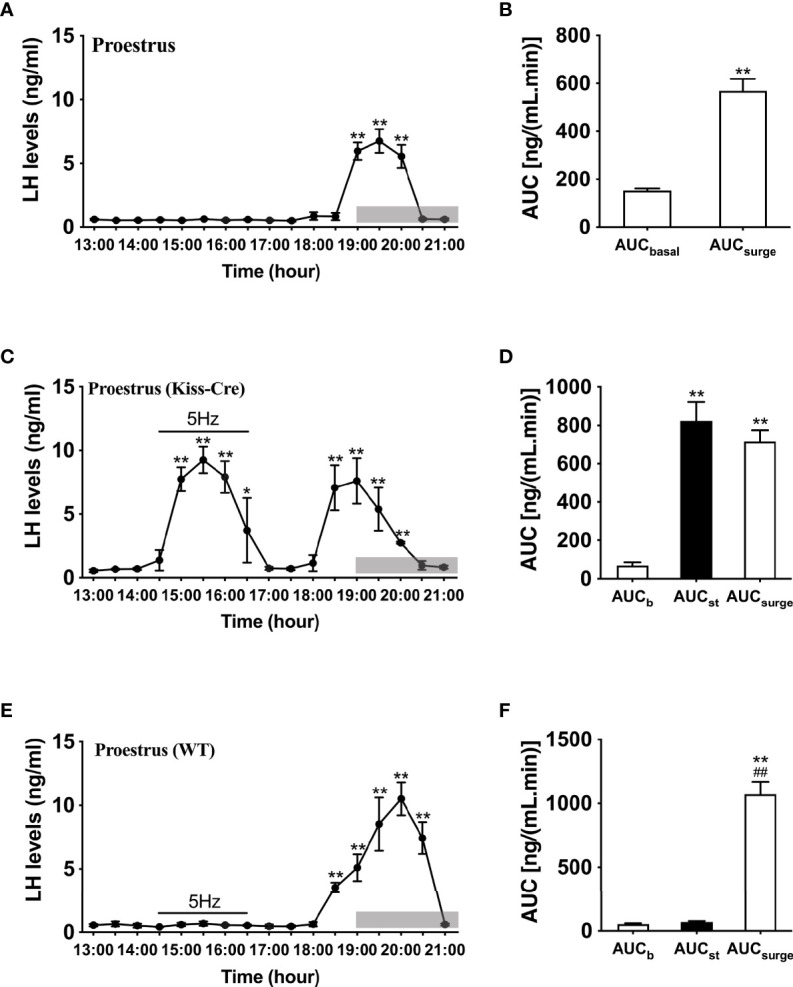
Spontaneous LH surge and LH surge-like response to optogenetic stimulation of ARC kisspeptin in proestrus Kiss-cre mice. **(A)** Circulating levels of LH (Mean ± SEM) in proestrus Kiss-cre mice sampled at 30-min intervals (13:00 – 21:00 h) showing spontaneous LH surges (n = 6). **(B)** Area under curve of LH levels for basal (AUC_b_, 13:00 - 17:30 h) and spontaneous LH surge (AUC_surge_, 18:30 - 20:30 h) time periods. **(C)** Evoked LH secretion in response to sustained 5 Hz blue-light stimulations of ARC kisspeptin neuron cell bodies (14:30 - 16:30 h) in Kiss-Cre mice (n = 5). **(D)** Area under curve of LH levels before stimulation (basal) (AUC_b_, 13:00 - 14:30 h), during stimulation (AUC_st_, 14:30 - 16:30 h) and during the spontaneous surge (AUC_Surge_, 18:00 – 20:30 h) (n = 5). **(E)** LH levels of wild type (WT) mice sampled at 30-min intervals and subjected to same optic stimulation protocol as Kiss-cre mice (n = 4). **(F)** Area under curve of LH levels before stimulation (AUC_b_, 13:00 - 14:30 h), during stimulation (AUC_st_, 14:30 - 16:30 h) and during the spontaneous surge (AUC_Surge_, 18:00 - 21:00 h). *P < 0.05 and **P < 0.01 (unpaired t-test) relative to basal LH levels or AUC for basal LH levels. ^##^P< 0.01 indicates a significant difference relative to AUC for stimulation period. Lights off is at 19:00 h for all experiments, as indicated by the grey bar on the abscissa.

Proestrus was identified by vaginal cytology. Proestrous LH surge profiles were examined in ovary intact mice. Of the eight proestrous mice, six showed an increase in LH secretion (**Figure 2A**), with the remainder having no change in LH levels from baseline during the sampling period (data not shown). The six proestrous mice exhibiting an LH surge displayed a surge onset at 18:30 h (**Figure 2A**) with a significant increase in area under the curve (AUC) during the surge time (**Figure 2B**). For these eight proestrous mice, the basal level prior to surge onset was 0.55 ± 0.01 ng/mL and the mean peak level of the LH surge was 5.80 ± 1.13 ng/mL. The effects of sustained stimulation for 2 h from 14:30 to 16:30 h (early stimulation) were examined during proestrus in in Kiss-Cre, as well as in wild type mice. Proestrus Kiss-Cre mice showed increased LH surge-like release during the early stimulation period, in addition to a spontaneous LH surge onset at 18:00 h (n = 5; **Figures 2C, D**). AUC during the stimulation and spontaneous LH surge period were comparable (**Figure 2D**). No response during optic stimulation was observed in wild type mice, but a spontaneous LH surge occurred at the usual time of day (n = 4, **Figures 2E, F**).

### Effect of Optogenetic Stimulation of ARC Kiss1 Neurons on LH Secretion in OVX Mice With Different Gonadal Steroids Replacement Regimes

**Figure 3:**
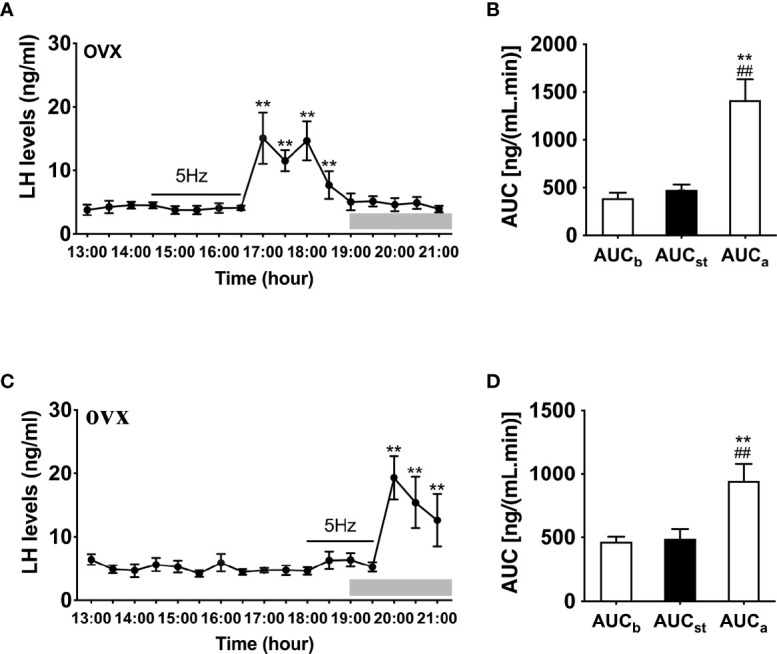
Effects of optogenetic activation of ARC kisspeptin neurons on LH secretion in OVX Kiss-cre mice. **(A)** Evoked LH secretion (Mean ± SEM) in response to 5 Hz blue-light stimulations of ARC kisspeptin neuron (14:30 - 16:30 h) (n = 5). **(B)** Area under curve of LH levels before (basal) (AUC_b_, 13:00 - 14:30 h), during (AUC_st_, 14:30 - 16:30 h) and after stimulation (AUC_a_, 16:30 – 18:30 h). **(C)** Evoked LH secretion in response to 5 Hz optic stimulations of ARC kisspeptin neuron from 18:00 to 19:30 h (n = 5). **(D)** Area under curve of LH levels before (AUC_b_, 13:00 - 14:30 h), during (AUC_st_, 18:00 - 19:30 h) and after stimulation (AUC_a_, 19:30 - 21:00 h). **P < 0.01 (unpaired t-test) relative to basal LH levels or AUC for basal LH levels. ^##^P < 0.01 indicates a significant difference relative to AUC for stimulation periods. Lights off is at 19:00 h for all experiments, as indicated by the grey bar on the abscissa.

We then examined the effects of sustained stimulation for 2 h from 14:30 to 16:30 h (early stimulation) and 1.5 h from 18:00 to 19:30 h (late stimulation) in OVX Kiss-Cre female mice. The optic stimulation of ARC Kiss1 neurons had no immediate effect on LH secretion in OVX mice (n = 5, **Figure 3A**). However, surprisingly LH secretion was delayed and significantly increased after terminating the stimulation. This finding was consistent in both the early and late stimulation groups (**Figures 3A, C**). The peak evoked LH levels for early (n = 5) and late (n = 5) activation were 18.67 ± 3.22 ng/mL and 25.75 ± 2.00 ng/mL, respectively. AUC for the rise in LH levels post stimulation offset are shown in **Figures 3B, D**.

**Figure 4:**
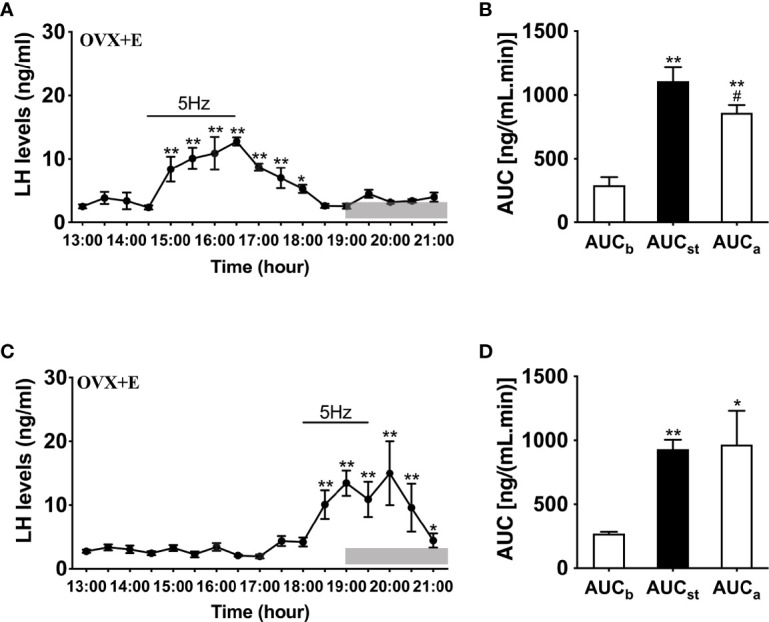
Effects of optogenetic activation of ARC kisspeptin neurons on LH secretion in OVX estradiol capsule implanted Kiss-cre mice. **(A)** Evoked LH secretion (Mean ± SEM) in response to 5 Hz blue-light stimulations of ARC kisspeptin neurons (14:30 - 16:30 h) in Kiss-cre OVX mice implanted with estradiol capsules (OVX+E) (n = 5). **(B)** Area under curve of LH levels before (AUC_b_, 13:00 - 14:00 h), during (AUC_st_, 14:30 - 16:30 h) and after optic stimulation (AUC_a_, 16:30 - 18:30 h). **(C)** Evoked LH secretion in response to 5 Hz optic stimulations of ARC kisspeptin neuron (18:00 -19:30 h) Kiss-Cre OVX+E mice (n = 4). **(D)** Area under curve of LH levels before (AUC_b_, 16:00 - 17:30 h), during (AUC_st_, 18:00 - 19:30 h) and after stimulation (AUC_a_, 19:30 - 21:00 h). *P < 0.05 and **P < 0.01 (unpaired t-test) relative to basal LH levels or AUC for basal LH levels. ^#^P < 0.05 indicates a significant difference relative to AUC for stimulation periods. Lights off is at 19:00 h for all experiments, as indicated by the grey bar on the abscissa.

To further determine whether LH release in response to optogenetic stimulation of ARC Kiss1 neurons is influenced by sex steroids *in vivo*, we implanted estradiol-containing capsules in OVX Kiss-Cre mice (OVX+E). Stimulation in the presence of diestrous levels of estradiol generated significant instantaneous increments in LH release during the stimulation for both early (n = 5) and late (n = 4) stimulation groups (**Figures 4A–D**); this lasted at least 2 h and was a consistent finding between the two groups.

**Figure 5:**
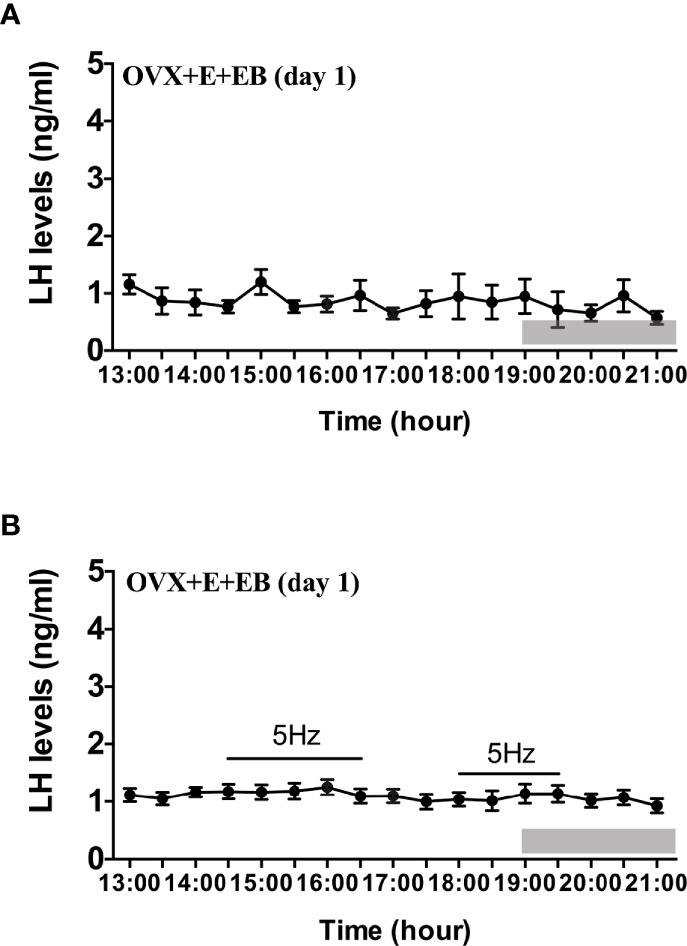
Effects of optogenetic activation of ARC kisspeptin neurons on LH secretion in OVX estradiol capsule implanted Kiss-cre mice injected with estradiol benzoate. **(A)** Mean circulating levels of LH without optic stimulations of ARC kisspeptin neurons in OVX Kiss-cre mice implantation of estradiol capsule (E) and injection of estradiol benzoate (EB) and blood samples collected on the day of EB injection (OVX+E+EB, day1) (n = 4). **(B)** Mean circulating levels of LH secretion in response to 5 Hz blue-light stimulations of ARC kisspeptin neuron (14:30 - 16:30 h and 18:00 - 19:30 h) in Kiss-cre OVX+E+EB mice (n = 5). The values are presented as mean ± SEM. Lights off is at 19:00 h for all experiments, as indicated by the grey bar on the abscissa.

**Figure 6:**
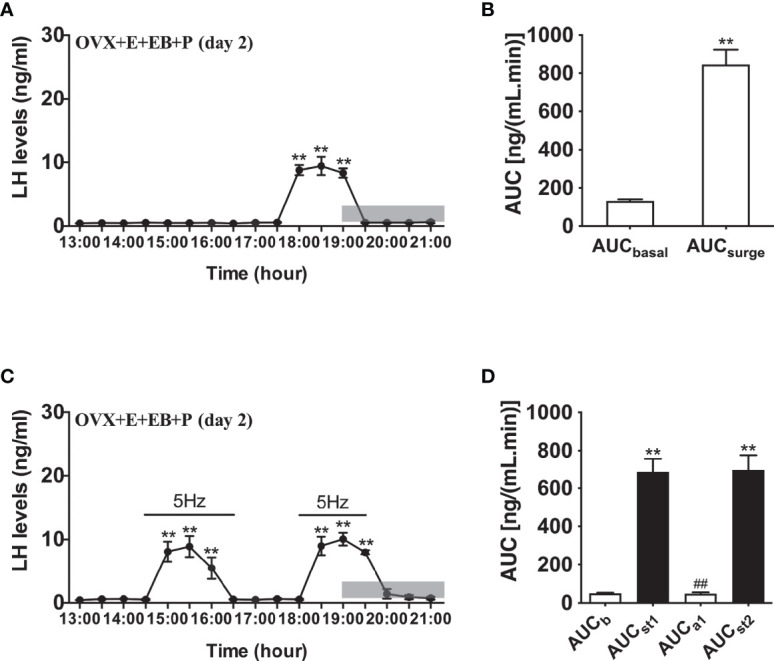
Effects of optogenetic activation of ARC kisspeptin neurons on LH secretion in OVX estradiol capsule implanted Kiss-cre mice injected with estradiol benzoate and subsequently progesterone. **(A)** Mean circulating levels of LH (mean ± SEM) without optic stimulations of ARC kisspeptin neurons in OVX Kiss-cre implanted with estradiol capsules (E) and injected with estradiol benzoate (EB) and the following day injected with progesterone (P) as an LH surge inducing protocol (OVX+E+EB+P) (n = 4). Note the expected steroid induced LH surge at 17:30 h. **(B)** Area under curve of LH levels for basal (AUC_b_, 13:00 - 17:30 h) and surge (AUC_surge_, 17:30 - 19:30 h) time periods. **(C)** Evoked LH secretion in response to 5 Hz optic stimulations of ARC kisspeptin neuron (14:30 - 16:30 h and 18:00 - 19:30 h) in Kiss-cre OVX+E+EB+P mice (n = 5). **(D)** Area under curve of LH levels before early stimulation (basal) (AUC_b_, 13:00 - 14:30 h), during first stimulation (AUC_st1_, 14:30 - 16:30 h), after first stimulation (AUC_a1_, 16:30 - 18:00 h), and during second stimulation (AUC_st2_, 18:00 - 19:30 h). The values are presented as mean ± SEM. **P < 0.01 (unpaired t-test) relative to basal LH levels or AUC for basal LH levels. ^##^P < 0.01 indicates a significant difference relative to AUC for LH levels during the first and second stimulation periods. Lights off is at 19:00 h for all experiments, as indicated by the grey bar on the abscissa.

Compared to OVX mice with only the estradiol capsule implanted, neither early or late optogenetic stimulation of ARC Kiss1 neurons led to an increase in LH secretion in OVX+E+EB mice (**Figure 5B**; n = 5), and as expected no spontaneous rise in LH occurred on the day of EB administration with the OVX+E+EB regime (**Figure 5A**; n = 4). However, after injection of progesterone on the next day for these mice (OVX+E+EB+P), all 4 animals not exposed to optic stimulation exhibited a spontaneous LH surge with a consistent onset at 18:00 h, approximately 1 h prior to lights off (**Figure 6A**; n = 4), with all values being significantly different from basal and the AUC during the surge time was higher than basal period (**Figure 6B**). In addition to the similar endogenous LH surges, the early and late optic stimulation generated a profile of LH surge-like secretion during the stimulation period without any delay and these were recovered immediately after terminating stimulation (**Figure 6C**, n = 4). The AUC showed the response of optogenetic activation directly in **Figure 6D**.

Taking these data together, the results demonstrate that 5 Hz activation of ARC Kiss1 neurons is remarkably effective at evoking an LH surge-like secretion in female mice with or without estradiol, although there is a markedly delayed response in mice without estradiol.

## Discussion

The results from the present study provide novel evidence that activation of ARC Kiss1 neurons can evoke surge-like increments in LH secretion, depending on the steroid milieu present. In intact mice, ARC Kiss1 neuron stimulation was sufficient to cause a surge-like increase in LH secretion whereas this response was present but delayed, i.e., occurring after ARC stimulation was terminated, in the absence of E in OVX mice. When OVX mice were implanted with an E capsule to mimic diestrous levels, the surge-like increase in LH returned during ARC Kiss1 neuron stimulation. On the day of EB injection in the OVX+E mice, the classical negative feedback stage, there was no LH response during optogenetic stimulation, however upon addition of progesterone the following day, a spontaneous LH surge was seen as expected, as well as the surge-like increases upon ARC Kiss1 neuron stimulation.

It has been well documented that the LH surge in rodents is tightly connected to the circadian system. Disruption of the circadian system by exposure to abnormal light-dark cycles or mutations in core clock genes has been shown to cause diminished reproductive capacity (23, 24). Administration of barbiturates in rodents during a “critical period” on the afternoon of proestrus has been shown to prevent the LH surge, indicating that a circadian neural trigger for surge occurs in this interval (25, 26). In OVX mice treated with the appropriate E regime, a relatively uniform LH surge was observed, with onset at approximately 0.5 h prior to lights off (21). Considering these findings, we have sampled from 13:00-21:00 h to examine the effect of ARC Kiss1 stimulation on LH secretion.

In order to determine whether the ARC is involved in modulating LH surges and whether this was dependent on the steroid hormonal milieu, we implanted an E-filled capsule in OVX female mice, which were then given a s.c. injection of EB or EB+P to investigate the LH surges after the optic stimulation. Interestingly, it is AVPV not ARC Kiss1 that has traditionally been associated with surge generation. Tracing studies suggest that ARC Kiss1 neurons do not actually have any direct contact with the cell bodies of GnRH neurons but are instead linked to them *via* surge-generating AVPV Kiss1 neurons (27, 28). Therefore, it would not be unreasonable to suggest that contact between these two neuronal populations contributes to surge generation. Indeed, knockdown of kisspeptin in the ARC has been shown to decrease both the LH pulse frequency and amplitude of LH surges (18). Furthermore, *in vitro* optogenetic studies have demonstrated functional excitatory glutamatergic inputs to AVPV Kiss1 neurons from ARC Kiss1 neurons (29). Not only are most ARC Kiss1 neurons glutamatergic (30), but their glutamate expression is increased by E, supporting an LH surge functionality (31). Given that NKB receptors are essentially absent from AVPV Kiss1 neurons (32), it is unlikely that potential NKB release within the AVPV region following ARC Kiss1 optic stimulation contributed the surge-like release of LH. These findings might suggest that ARC Kiss1 neurons participate in the generation of LH surge by facilitating glutamate release from their neuronal projections onto the AVPV Kiss1 neurons, which in turn drive the GnRH neurons. However, further work is required to examine this postulate.

In OVX mice receiving no hormonal replacement, ARC stimulation induced the release of LH in a delayed manner, leading to a surge-like pattern of LH secretion after the stimulation period. Lack of E replacement may alter neuronal membrane potentials (33) which may mean reaching the threshold for kisspeptin firing leading to LH release required significant stimulation and did not occur during our stimulation period. This is further supported by the fact the estradiol has been shown to increase the sensitivity of kisspeptin on downstream neurons (34); however further work will need to be done to explain the effects we have seen upon ARC stimulation. Nevertheless, the lack of an immediate effect of optic stimulation of ARC Kiss1 neurons on LH secretion in OVX mice receiving no hormonal replacement would argue against a probable direct effect of on the GnRH dendron (35) to explain the LH surge-like responses that were observed in the present study.

In our study, we found that stimulating ARC Kiss1 in gonadal intact mice was enough to generate an LH surge-like profile outside of the usual surge time and of comparable magnitude. This is consistent with the postulate that ARC Kiss1 neurons may have synergistic and amplificatory roles in LH surge generation. Similarly, OVX+E mice displayed a surge-like LH pattern during stimulation. This was demonstrated when stimulation occurred in the early afternoon as well as during stimulation at the expected surge time. It is widely accepted that positive feedback leading to surge generation requires persistently high elevated levels of E; however, this was not the hormonal milieu used here. The fact that ARC stimulation is sufficient to cause a surge-like pattern in *low* E conditions, typical of diestrous, further supports the postulate that the ARC has roles in amplifying the LH surge.

On the first day of EB injection in OVX+E mice (day 1), there was no spontaneous LH surge, as expected, but no response during ARC stimulation. This is likely because surge generation requires persistently elevated levels of E not just an instantaneous elevation which we have used here. Our result was consistent with previous studies which reported that the treatment of EB alone for one day was not enough to generate a robust LH surge (36, 37). Experiments in rats have indicated 7-12h period is the absolute minimum time for estradiol to exert its facilitatory effect on the gonadotrophin response (38). However, upon injection of P the following day (day 2), spontaneous surges were restored. Surge-like LH secretion was also observed during ARC stimulation. Addition of P to an already “estradiol-primed” environment has been suggested as the true trigger behind LH surges (23) which explains the occurrence of the spontaneous LH surge in this model. ARC stimulation in this hormonal environment was enough to cause a surge-like pattern of LH secretion in both the early afternoon as at the expected surge time; again, being consistent with the hypothesis that ARC Kiss1 neurons may have synergistic and amplificatory roles in LH surge generation.

The AVPV Kiss1 neurons are thought to not only integrate hormonal but circadian signals to regulate the timing of the preovulatory LH surge. There is clear evidence that inputs from the circadian master clock, the suprachiasmatic nucleus (SCN), including vasopressin neurons, to the AVPV Kiss1 neurons are responsible for mediating the time-of-day dependent surge (39). Interestingly, ARC Kiss1 neurons were recently shown to project heavily to the hypothalamic subparaventricular zone (SPZ), a major relay hub of the SCN involved in orchestrating circadian rhythmicities such as sleep and locomotion, whilst avoiding the SCN per se (28, 40). Indeed, silencing of ARC Kiss1 neurons resulted in dysregulation of circadian rhythms including sleeping, feeding and activity (40). Moreover, SCN vasopressin efferent projections to the SPZ appears to be contacted by ARC Kiss1 fiber projections (28), which raises the possibility that ARC Kiss1 neurons may impact on SCN vasopressin signaling to AVPV Kiss1 neurons, which has been shown to be critically dependent on circulating ovarian steroids (39), to regulate LH surge expression.

It is important to point out that while the majority, approximately 85%, of the Cre-expressing neurons have previously been shown to contain Kiss1 immunoreactivity in the mouse model used in the present study (20), without the validation of EYFP-Kiss1 colocalization the role of the potentially remaining 15% of non-Kiss1 EYFP-infected neurons remains a caveat to the interpretation of the results presented. It is also important to point out that in the non-rodent species, the ARC KNDy neurons are considered the major population involved in GnRH/LH surge generation (41, 42). Therefore, we must acknowledge the caveat that in non-rodent species, there may be no amplificatory role for these neurons as demonstrated in the rodent species.

In conclusion, we have used selective optogenetic stimulation of Kiss1 neurons specifically in the ARC nucleus and found that activation of these neurons under different sex steroid milieus has the ability to evoke surge-like patterns in LH secretion. Taken together, these results help to affirm the fact that ARC Kiss1 may have a novel amplificatory role in LH surge production.

## Data Availability Statement

The original contributions presented in the study are included in the article/supplementary material. Further inquiries can be directed to the corresponding author.

## Ethics Statement

The animal study was reviewed and approved by the Animal Welfare and Ethical Review Body Committee at King’s College London.

## Author Contributions

KO’B and X-FL conceived the study. KO’B, X-FL, and H-FH contributed to the design of the study. X-HL, GL, and HW contributed to animal experiment. X-HL, GL, and L-SK contributed to data collection. All authors contributed to the article and approved the submitted version.

## Funding

Grants supporting paper: UKRI: BBSRC (BB/S000550/1) and MRC (MR/N022637/1); the National Key Research and Development Program of China (2018YFC1005001 to X-HL); the National Natural Science Foundation of China (82071730 to X-HL). X-HL is a KC Wong Postdoctoral Fellow.

## Conflict of Interest

The authors declare that the research was conducted in the absence of any commercial or financial relationships that could be construed as a potential conflict of interest.

## Publisher’s Note

All claims expressed in this article are solely those of the authors and do not necessarily represent those of their affiliated organizations, or those of the publisher, the editors and the reviewers. Any product that may be evaluated in this article, or claim that may be made by its manufacturer, is not guaranteed or endorsed by the publisher.
